# Rare and low-frequency exonic variants and gene-by-smoking interactions in pulmonary function

**DOI:** 10.1038/s41598-021-98120-7

**Published:** 2021-09-29

**Authors:** Tianzhong Yang, Victoria E. Jackson, Albert V. Smith, Han Chen, Traci M. Bartz, Colleen M. Sitlani, Bruce M. Psaty, Sina A. Gharib, George T. O’Connor, Josée Dupuis, Jiayi Xu, Kurt Lohman, Yongmei Liu, Stephen B. Kritchevsky, Patricia A. Cassano, Claudia Flexeder, Christian Gieger, Stefan Karrasch, Annette Peters, Holger Schulz, Sarah E. Harris, John M. Starr, Ian J. Deary, Ani Manichaikul, Elizabeth C. Oelsner, R. G. Barr, Kent D. Taylor, Stephen S. Rich, Tobias N. Bonten, Dennis O. Mook-Kanamori, Raymond Noordam, Ruifang Li-Gao, Marjo-Riitta Jarvelin, Matthias Wielscher, Natalie Terzikhan, Lies Lahousse, Guy Brusselle, Stefan Weiss, Ralf Ewert, Sven Gläser, Georg Homuth, Nick Shrine, Ian P. Hall, Martin Tobin, Stephanie J. London, Peng Wei, Alanna C. Morrison

**Affiliations:** 1 Department of Biostatistics and Data Science, School of Public Health, The University of Texas Health Science Center at Houston, Houston, TX USA; 2 Division of Biostatistics, School of Public Health, University of Minnesota, Minneapolis, MN USA; 3 Department of Health Sciences, University of Leicester, Leicester, UK; 4 Department of Biostatistics, University of Michigan, Ann Arbor, MI USA; 5 Human Genetics Center, Department of Epidemiology, Human Genetics and Environmental Sciences, School of Public Health, The University of Texas Health Science Center at Houston, Houston, TX USA; 6 Center for Precision Health, School of Public Health and School of Biomedical Informatics, The University of Texas Health Science Center at Houston, Houston, TX USA; 7 Department of Biostatistics, University of Washington, Seattle, USA; 8 Cardiovascular Health Research Unit, Department of Medicine, University of Washington, Seattle, WA USA; 9 Cardiovascular Health Research Unit, Departments of Medicine, Epidemiology and Health Services, University of Washington, Seattle, WA USA; 10 Kaiser Permanente Washington Health Research Institute, Seattle, WA USA; 11 Division of Pulmonary, Critical Care, and Sleep, Department of Medicine, University of Washington, Seattle, WA USA; 12 Department of Medicine, Pulmonary Center, Boston University School of Medicine, Boston, MA USA; 13 National Heart, Lung, and Blood Institute’s Framingham Heart Study, Framingham, MA USA; 14 Department of Biostatistics, Boston University School of Public Health, Boston, MA USA; 15 Pamela Sklar Division of Psychiatric Genomics, Department of Genetics and Genomic Sciences, Icahn School of Medicine at Mount Sinai, New York, NY USA; 16 Division of Nutritional Sciences, Cornell University, Ithaca, NY USA; 17 Division of Cardiology, Department of Medicine, Duke Molecular Physiology Institute, Duke University School of Medicine, Durham, NC USA; 18 Sticht Center for Healthy Aging and Alzheimer’s Prevention, Wake Forest School of Medicine, Winston-Salem, NC USA; 19 Department of Healthcare Policy and Research, Weill Cornell Medical College, New York, NY USA; 20 Institute of Epidemiology, Helmholtz Zentrum München, German Research Center for Environmental Health, Neuherberg, Germany; 21 Research Unit of Molecular Epidemiology, Institute of Epidemiology, Helmholtz Zentrum München, German Research Center for Environmental Health, Neuherberg, Germany; 22 Institute and Outpatient Clinic for Occupational, Social and Environmental Medicine, Ludwig-Maximilians-Universität, Munich, Germany; 23 Comprehensive Pneumology Center Munich (CPC-M), German Center for Lung Research (DZL), Munich, Germany; 24 Institute for Medical Information Processing, Biometry and Epidemiology, Ludwig-Maximilians-Universität München, Munich, Germany; 25 Department of Psychology, The University of Edinburgh, Edinburgh, UK; 26 Centre for Cognitive Ageing and Cognitive Epidemiology, The University of Edinburgh, Edinburgh, UK; 27 Alzheimer Scotland Dementia Research Centre, The University of Edinburgh, Edinburgh, UK; 28 Department of Public Health Sciences, Center for Public Health Genomics, University of Virginia, Charlottesville, VA USA; 29 Division of Biostatistics and Epidemiology, Department of Public Health Sciences, University of Virginia, Charlottesville, VA USA; 30 Department of Medicine, College of Physicians and Surgeons, Columbia University, New York, NY USA; 31 Department of Epidemiology, Mailman School of Public Health, Columbia University, New York, NY USA; 32 Department of Medicine, Department of Epidemiology, Columbia University Medical Center, New York, NY USA; 33 David Geffen School of Medicine, University of California, Los Angeles, Los Angeles, CA USA; 34 Department of Public Health Sciences, Center for Public Health Genomics, University of Virginia, Charlottesville, VA USA; 35 Department of Public Health and Primary Care, Leiden University Medical Center, Leiden, The Netherlands; 36 Department of Clinical Epidemiology, Leiden University Medical Center, Leiden, The Netherlands; 37 Department of Public Health and Primary Care, Leiden University Medical Center, Leiden, The Netherlands; 38 Division of Gerontology and Geriatrics, Department of Internal Medicine, Leiden University Medical Center, Leiden, The Netherlands; 39 Department of Epidemiology and Biostatistics, School of Public Health, Imperial College London, London, UK; 40 Faculty of Medicine, Center for Life Course Health Research, University of Oulu, Oulu, Finland; 41 Biocenter of Oulu, University of Oulu, Oulu, Finland; 42 Department of Epidemiology, Erasmus University Medical Center, Rotterdam, The Netherlands; 43 Department of Respiratory Medicine, Ghent University Hospital, Ghent, Belgium; 44 Interfaculty Institute for Genetics and Functional Genomics, University Medicine Greifswald, Greifswald, Germany; 45 DZHK (German Centre for Cardiovascular Research), Partner Site Greifswald, Greifswald, Germany; 46 Division of Cardiology, Pneumology, Infectious Diseases, Intensive Care Medicine, Department of Internal Medicine B, University Medicine Greifswald, Greifswald, Germany; 47 Department of Internal Medicine, Vivantes Hospital Berlin Spandau, Berlin, Germany; 48 Division of Respiratory Medicine, University of Nottingham, Nottingham, UK; 49 Leicester Respiratory Biomedical Research Unit, National Institute for Health Research, Glenfield Hospital, Leicester, UK; 50 Department of Health and Human Services, Epidemiology Branch, National Institute of Environmental Health Sciences, National Institutes of Health, Research Triangle Park, NC USA; 51 Department of Biostatistics, The University of Texas MD Anderson Cancer Center, Houston, TX USA

**Keywords:** Genetics, Diseases

## Abstract

Genome-wide association studies have identified numerous common genetic variants associated with spirometric measures of pulmonary function, including forced expiratory volume in one second (FEV_1_), forced vital capacity, and their ratio. However, variants with lower minor allele frequencies are less explored. We conducted a large-scale gene-smoking interaction meta-analysis on exonic rare and low-frequency variants involving 44,429 individuals of European ancestry in the discovery stage and sought replication in the UK BiLEVE study with 45,133 European ancestry samples and UK Biobank study with 59,478 samples. We leveraged data on cigarette smoking, the major environmental risk factor for reduced lung function, by testing gene-by-smoking interaction effects only and simultaneously testing the genetic main effects and interaction effects. The most statistically significant signal that replicated was a previously reported low-frequency signal in *GPR126*, distinct from common variant associations in this gene. Although only nominal replication was obtained for a top rare variant signal rs142935352 in one of the two studies, interaction and joint tests for current smoking and *PDE3B* were significantly associated with FEV_1_. This study investigates the utility of assessing gene-by-smoking interactions and underscores their effects on potential pulmonary function.

## Introduction

Measures of pulmonary function provide important clinical and research tools for evaluating lung disease and other morbidities^1^. Pulmonary function is used to diagnose chronic obstructive pulmonary disease and to monitor the severity and progression of many lung conditions. Furthermore, even within the normal range, pulmonary function is a risk factor for mortality and morbidity from various conditions^2–5^. Genome-wide association studies (GWASs) have identified many single nucleotide variants (SNVs) for pulmonary function after adjusting for cigarette smoking, a well-established risk factor for reduced pulmonary function, as a confounder and potential effect modifier^6–14^. The interplay between genetic and environmental factors likely plays a role in complex traits, including chronic lung diseases. A large GWAS meta-analysis has shown the existence of SNV-by-smoking interaction in relation to two measures of pulmonary function (forced expiratory volume in one second, FEV_1_, and the ratio of FEV_1_ to the forced vital capacity, FEV_1_/FVC) by performing joint analyses of genetic main effects and interaction effects of single SNVs and their interaction with smoking^14^. However, there is a lack of gene-by-environment (GxE) interaction analysis involving low-frequency (minor allele frequency [MAF] 1% to 5%) or rare variants (MAF less than 1%)^15^, which are typically not well captured or imputed by previous GWAS meta-analyses of pulmonary function. The more recent use of genotyping platforms including lower frequency and rare variants enables better assessment of the role of rare genetic variation in disease.

Due to the low power to detect association with rare variants, a commonly adopted strategy in analyzing rare variant-phenotype associations is to group variants according to genes or genomic regions and test whether the grouped variants are associated with the phenotype^16^. While single-variant-based interaction tests for common variants are well-established^17,18^, methods for detecting rare variant GxE interactions are relatively new. Recently developed novel approaches for testing rare variant GxE interaction effects include a joint test that allows for simultaneous testing of the genetic main and interaction effects as well as the ability to assess gene-based GxE interactions only for both related and unrelated individuals^19–22^. The joint test and the test for the interaction terms only are both important for different scientific hypotheses. The joint test assesses genetic associations with pulmonary function, accounting for heterogeneity of genetic effects in individuals with different environmental exposures (in our context, smoking behaviors). The test of the interaction terms only can identify genes that modify the effect of smoking on pulmonary function. For single variant analysis, an interaction requires at least four fold larger sample size than a genetic main effect of comparable magnitude^23^. Similarly, for gene-based interaction tests, larger sample sizes are required for detection, especially for rare variants. This study is the first to incorporate GxE interactions in modeling rare and low-frequency genetic variants and smoking effects on pulmonary function based on large samples. In discovery, we used genetic and phenotypic information from the Cohorts for Heart and Aging Research in Genomic Epidemiology (CHARGE)^24^ and SpiroMeta Consortia, and we sought replication of findings in the UK BiLEVE and UK Biobank studies.

## Results

Characteristics and sample sizes of the study cohorts are presented in Table 1. We focused on two smoking categories throughout: ever-smokers vs never-smokers and current smokers vs never-smokers and past-smokers combined. The percentage of ever-smokers per study ranged from 30 to 61% in 44,429 European Ancestry participants and the percentage of current smokers per study ranged from 6 to 25%.

**Table 1:** Descriptive statistics for each participating cohort.

Study	Sample size	Male (%)	Age (sd)	Ever smokers (%)	Current smoker (%)	FEV_1_ (sd)	FVC (sd)	Ratio (sd)
**Discovery stage**								
ARIC	10,874	46.8	54.3 (5.7)	40.3	24.3	2935.4 (768.1)	3977.2 (978.0)	73.8 (7.6)
FHS	6172	46.6	44.9 (10.8)	47.8	19.1	3325.6 (844)	4321.9 (1043.2)	77.0 (7.0)
1958BC	4594	55.7	42 (0)	53.4	24.9	3350.0 (782.74)	4275.0 (1024.6)	78.9 (8.4)
RS	567	54.7	79.9 (5.0)	30.0	9.9	2265.68 (679.79)	3025.1 (860.3)	75.0 (8.0)
SHIP	4694	49.3	49.9 (14.4)	39.3	24.6	3260.9 (905.6)	4021.7 (1087.5)	81.2 (6.9)
NFBC1966	1431	45.6	31 (0)	53.0	25.6	3925.1 (778.1)	4685.2 (989.1)	84.2 (6.1)
NEO	6203	47.5	55.7 (6.0)	35.6	16.2	3239.8 (795.4)	4222.8 (1025.1)	77.0 (6.7)
CHS	3496	43.3	72.6 (5.5)	43.5	10.1	2114.8 (653.3)	3026.2 (842.0)	69.8 (10.1)
AGES	1459	40.9	76.3 (8.8)	45.1	11.9	2146.1 (676.0)	2883.8 (845.1)	74.1 (8.8)
LBC1936	970	50.4	69.5 (0.8)	47.2	10.7	2370.7 (676.1)	3040.6 (861.8)	78.5 (10.0)
KORA	1217	46.8	51.6 (5.7)	60.5	24.2	3341.7 (822.0)	4307.8 (1014.2)	77.6 (6.2)
MESA	1298	49.4	66.0 (9.8)	43.6	7.6	2565.5 (763.59)	3510.9 (993.34)	73.3 (8.7)
HABC	1454	53.2	73.7 (2.8)	56.5	6.5	2313.7 (653.5)	3114.5 (812.4)	73.7 (2.8)
**Replication stage**								
UK BiLEVE	45,133	49.1	57.0 (7.9)	49.3	18.2	3565.0 (851.7)	3573.0 (1040.1)	74.0 (7.3)
UK Biobank	59,478	43.2	56.0 (8.0)	45.0	8.5	2874.0 (725)	3754 (932.1)	76.7 (5.6)

FEV_1_ and FVC are in unit of mL.

Quantile–quantile plots of the meta-analysis results for the interaction and joint tests from the six combinations of smoking variables status (ever smoking or current smoking) and pulmonary function measures (FEV_1_, FVC, and FEV_1_/FVC ratio) of discovery stage are presented in Figure S1. Some quantile–quantile plots suggest inflation in the gene-by-current smoking interaction analyses, which could be due to the low frequency of current smokers. The few studies with larger inflation factors (λ > 1.5) in testing interactions between current smoking status and a specific lung function measure were not included in the corresponding meta-analysis of this smoking-trait combination (Table S1).

In the discovery-stage meta-analyses, *GPR126* was significantly associated with FEV_1_/FVC in the gene-by-current-smoking (*p*-value = 1.9E−09) and gene-by-ever-smoking analyses (*p*-value = 2.8E−08) based on joint tests. In both replication studies, *GPR126* was also statistically significant in the joint tests for gene-by-current-smoking (*p*-value = 1.1E−16 in UK BiLEVE, 1.4E−30 in UK Biobank) and gene-by-ever-smoking (*p*-value = 1.7E−16 in UK BiLEVE, 5.2E−29 in UK Biobank, Table 2). The average number of rare variants in *GPR126* across studies was 18.8 (range from 5 to 25). The *p*-value of the interaction test was not significant for either smoking exposure evaluated (*p*-value > 0.5), suggesting that these joint test results were largely due to the genetic main effects.

**Table 2:** Significant genes in the meta-analyses (significant threshold = 5.7E−07).

Gene	Chr	Smoking status and phenotype Combination	Average # variants across studies (range)	Discovery Stage		Replication Stage (UK BiLEVE)		Replication Stage (UK Biobank)		GTEx
				*P*-value (interaction)	*P*-value (joint)	*P*-value (interaction)	*P*-value (joint)	*P*-value (interaction)	*P*-value (joint)	Expressed in lung
*GPR126*	6	Current & Ratio	18.8 (5–25)	0.40	1.9E−09	0.61	1.1E−16	0.54	1.4E−30	Yes
		Ever & Ratio		0.91	2.8E−08	0.98	1.7E−16	0.39	5.2E−29	Yes
*PDE3B*	11	Current & FEV_1_	7.2 (4–9)	7.1E−06	2.9E−07	0.94	0.37	0.25	0.44	Yes

A common intronic variant rs3817928 (MAF = 22%) in *GPR126* was previously found to be significantly associated with FEV_1_/FVC among European ancestry^25^. Conditioning on this common variant rs3817928, the joint test of gene-by-current-smoking analysis for *GPR126* remained nominally significant in the discovery (*p*-value = 2.4E−03) and genome-wide significant in the replication studies (*p*-value = 3.9E−09 in UK BiLEVE and *p*-value = 9.6E−17 in UK Biobank, Table 3). The joint test of gene-by-ever-smoking analysis conditioning on rs3817928 was also nominally significant in the discovery stage and genome-wide significant in the replication stage (*p*-value = 0.01 in discovery stage, *p*-value = 4.3E−09 in UK BiLEVE, and *p*-value = 2.5E−16 in UK Biobank, Table 3).

**Table 3:** Conditional analysis of *GPR126* (adjusting for a common variant rs3817928).

Gene /variant	Smoking status and phenotype Combination	Discovery Stage		Replication Stage (UK BiLEVE)		Replication Stage (UK Biobank)	
		*P*-value (interaction)	*P*-value (joint)	*P*-value (interaction)	*P*-value (joint)	*P*-value (interaction)	*P*-value (joint)
**Conditional gene-based analysis**							
*GPR126*	Current & Ratio	0.19	2.44E−03	0.52	3.90E−09	0.77	9.58E−17
	Ever & Ratio	0.89	1.13E−02	0.97	4.26E−09	0.47	2.47E−16
**Conditional single-variant analysis on the top signal in *GPR126***							
rs17280293	Current & Ratio	0.66	3.93E−03	0.46	3.94E−09	0.68	9.31E−17
	Ever & Ratio	0.98	6.10E−03	0.97	3.97E−09	0.89	2.44E−16

To further determine whether any single low-frequency or rare variant may be driving the aggregate variant test results, we conducted single variant interaction only and joint test analyses for all the SNVs in *GPR126* and report the most significant SNV, rs17280293 (MAF = 3%), in Table 4. Figures S2 and S3 show the regional association plots for *GPR126*. The significant joint test results for rs17280293 were replicated in the UK BiLEVE and UK Biobank studies (Table 4). This SNV is predicted to be deleterious by SIFT score (score = 0.001) in dbNSFP^26^.

**Table 4:** Single variant analysis for the identified significant genes.

Variants	Gene	Chr	INFO	MAF (%)	Combination	Discovery Stage		Replication Stage (UK BiLEVE)		Replication Stage (UK Biobank)	
						*P*-value (interaction)	*P*-value (joint)	*P*-value (interaction)	*P*-value (joint)	*P*-value (interaction)	*P*-value (joint)
rs17280293	*GPR126*	6	1	2.8	Current & Ratio	0.74	8.0E−06	0.52	1.2E−16	0.50	1.5E−30
					Ever & Ratio	0.98	2.8E−05	0.99	1.6E−16	0.97	5.3E−29
rs142935352	*PDE3B*	11	0.98	0.6	Current & FEV_1_	5.8E−03	0.011	0.01	0.03	0.88	0.99
rs61736639	*PDE3B*	11	0.96	0.7	Current & FEV_1_	3.5E−03	1.7E−05	0.71	0.10	0.84	0.56

SNV rs17280293 is in low linkage disequilibrium (LD) with the previously reported common variant rs3817928 in *GPR126* (R^2^ = 0.13 based on 1000 Genomes database Phase 3^27^), thus rs17280293 remained highly significant in the single-variant-based analysis when conditioning on rs3817928 (Table 3). Recently, it was shown that the rs17280293 was the leading signal that drove a gene-based association between *GPR126* and FEV_1_/FVC when studying genetic main effects^15^. It was also found to be the most significant signal in the *GPR126* region associated with diffusing capacity of the lung^28^. Our analyses on the interaction-only and joint analyses further highlighted the importance of this low-frequency variant.

*PDE3B* was significantly associated with FEV_1_ in the interaction-only test (*p*-value = 1.9E−06) and joint test (*p*-value = 2.9E−07) in the gene-by-current-smoking analyses (Table 2). The *p*-value of the interaction test was highly significant, suggesting that the joint test result was at least partially driven by the interaction effects. However, the interaction effect was not replicated in either UK BiLEVE or UK Biobank. Single-variant-based interaction and joint analyses were performed on the variants within *PDE3B* (see the regional association plots in Figures S4 and S5). The top two variants are present in Table 4, among which variant rs142935352 (MAF = 0.6%) in *PDE3B* had a significant SNV-by-current smoking interaction in relation to FEV_1_ in the discovery studies and was nominally significant in the UK BiLEVE replication study for both the interaction-only (*p*-value = 0.01) and joint tests (*p*-value = 0.02). The other top variant rs61736639 (MAF = 0.7%) was not significant in the replication datasets. According to the Genotype-Tissue Expression (GTEx) project (^29^, accessed on 07/18/2021) both *GPR126* (median transcripts per million, TPM = 20.2) and *PDE3B* (median TPM = 8.8) were expressed in lung tissue, relative to a minimal expression threshold of TPM > 0.1^30^.

## Discussion

Most genome-wide analyses of pulmonary function have focused on common variants or genetic main effects^25,31^. However, the gene-level association could be missed if the signal is driven by rare variants or there is interaction with environmental exposures. Therefore, we focused on rare and low-frequency exonic genetic variants and their interaction with cigarette smoking, the major environmental risk factor for reduced lung function. Specifically, we performed gene-based tests of GxE interaction only and joint tests assessing genetic main effects and interaction effects. The joint analysis has been shown to be nearly optimal across a variety of models for common variants^17^. In some scenarios, the GxE interaction only tests could have higher statistical power than the joint tests^19,21^. To boost the statistical power for discovery, we combined data across thirteen studies with 44,429 European ancestry individuals through meta-analyses. Significant genes were examined in two replication studies with a total of 104,611 European ancestry individuals. This study is the first and the largest to incorporate GxE interactions in modeling rare and low-frequency variant genetic and smoking effects on pulmonary function. However, statistical power would continue to be improved with increased sample size. Table S3 and S4 provide the top 5 genes for the interaction only and joint tests, which may include the putative genes that did not reach genome-wide significance in the study.

Our results for the GxE joint analysis support the involvement of rare variants in a locus, *GPR126*. We identified one low-frequency variant rs17280293 which was successfully replicated in our study and was previously reported by Jackson et al^15^ in a genetic main effect analysis. The SNV is in low LD with the known common variant, suggesting a distinct role of the rare variants. Neither gene-based nor single variant tests identified interaction with smoking for this SNV or this gene. Additionally, we found that *PDE3B* was significantly associated with FEV_1_ in the GxE interaction and joint association analysis in the discovery studies, however neither the interaction or joint effect gene-based tests replicated, although one leading rare variant rs142935352 reached nominal significance in UK BiLEVE for the interaction and joint tests. *PDE3B* had not been reported to be associated with lung function but is believed to be an important regulatory factor in modulating the inflammatory response and expressed in the lung^32^. One possible explanation is that smoking increases the inflammatory response in the lung, leading to the gene-by-smoking interaction. The signal of *PDE3B* in the discovery dataset was mainly driven by two SNVs (rs142935352 and rs61736639), both quite rare (MAF < 0.5% in EA), thus difficult to replicate even with large samples. We found that it was challenging to replicate rare signals in general, as rare variants varied across different studies. A variant present in one study was likely not included in the other study (for example, being monomorphic or having high missing rates).

Our study has limitations. First, the results were obtained from European ancestry populations, although we recognized the importance of conducting multi-ancestry studies to identify novel association signals^31^. Second, a few quantile–quantile plots suggested inflation in the gene-by-current-smoking interaction and joint test statistics (Figure S1). GxE analysis is well-known to be prone to systematic inflation for common variants and there could be various factors driving the inflation^33–35^. It is not surprising to see such inflation in rare-variant-based GxE analysis. We speculate that the inflation occurred in some studies due to a combination of a low proportion of current smokers and relatively small sample size, where the asymptotic property of the test statistics may not hold. For example, LBC1936 with a sample size of 970 and 11% of current smokers had an inflation factor > 1.5 for gene-by-current smoking interaction tests but not gene-by-ever smoking interaction (Table S1). Although we took a number of steps to reduce the inflation (adjusting for principal components, excluding studies with large inflation factors, excluding phenotypic outliers followed by inverse normal transformation of the spirometric measures of pulmonary function, excluding a study for a particular gene if it had low minor allele count (< 20), and using dichotomized smoking risk factors^21^), there could still be some inflation. Third, we examined exonic rare variants, but we note that signals from rare variants in the regulatory regions are likely to interact with smoking. Inclusion of rare regulatory variants could improve our understanding of disease mechanisms and warrants further investigation. Fourth, our analysis did not take into account the type of cigarette smoking, which could increase the difficulty of replication due to the potential heterogeneity of GxE interaction effects. Fifth, rare and low-frequency variants in the two replication datasets UK BiLEVE and Biobank Study were imputed using the 1000 Genomes and UK10K haplotype reference panel^36^. Although the imputation quality had been examined through a pseudo-GWAS simulation^36^, it may still be suboptimal comparing with sequencing data, thus increasing the replication difficulty. In our case, SNVs included in *GPR126* and *PDE3B* all had INFO score > 0.8 and the leading SNVs had INFO score > 0.95 (Table 4), thus less likely to be affected by the genotyping array platform.

The strengths of the study include the relatively large sample size with genotyping of rare and low-frequency variants. In addition, we employed recently developed methods for incorporating environmental interactions in the study of the influence of rare variants in disease to decrease the multiple testing burden, potentially increase the statistical power. We found that one of the first discovered genes associated with pulmonary function in GWAS of common variants, *GPR126*, likely has low-frequency variants contributing to its role in this phenotype and other putative genes. The gene was successfully replicated but the signal may be driven by the genetic main effect.

## Materials and methods

### Ethics statement

All the studies contributed to our analyses have their protocols approved by the respective local Institutional Review Board with the details in the Supplementary Materials. All participants wrote informed consent for the genetic studies and all experiments were performed in accordance with relevant guidelines and regulations.

### Cohort studies

We combined data from 13 studies for the discovery stage, either from the CHARGE Consortium or the SpiroMeta Consortium: the Age, Environment, Susceptibility (AGES) study, Atherosclerosis Risk in Communities (ARIC) study^37^, British 1958 Birth Cohort (1958BC)^38^, Cardiovascular Health Study (CHS)^39^, Framingham Heart Study (FHS)^40,41^, Health, Aging, and Body Composition (HABC) study^42^, Northern Finland Birth Cohort of 1966 (NFBC1966)^43^, Multi-Ethnic Study of Atherosclerosis (MESA)^44^, Rotterdam Study (RS)^45^, Study of Health in Pomerania (SHIP)^46^, the Netherlands Epidemiology of Obesity Study (NEO)^47^, Lothian in Birth Cohort 1936 (LBC1936) study^48^, and Cooperative Health Research in the Region of Augsburg (KORA) study^49^. The discovery studies consisted of 44,429 individuals. Replication was conducted in two studies: UK BiLEVE Study (n = 45,133) and UK Biobank (n = 59,478)^50^. Study descriptions are provided in the Supplementary Note of Jackson et al., 2018^15^ and Artigas et al., 2015^51^.

### Pulmonary function measurements and smoking information

Spirometric measures included FEV_1_, FVC, and their ratio (FEV_1_/FVC). Details of the spirometry have been previously reported for the discovery^15^ and replication studies^52^. Because outlier phenotype values can be influential in rare variant analyses, pulmonary function values with residuals after regressing out covariates (listed below) on each lung function trait larger than four standard deviations from the mean were examined by investigators from each cohort expert in the collection of pulmonary function data. Data points were evaluated regarding whether they were biologically plausible for that individual as opposed to more consistent with data errors. To ensure the normality of the FEV_1_, FVC, and FEV_1_/FVC, a ranked-based inverse-normal transformation was performed on each pulmonary function trait by the individual participating studies. For FEV_1_/FVC, the ratio was first calculated prior to transformation.

Cigarette smoking can have important adverse effects on pulmonary function. Smoking exposure was ascertained by questionnaire at the same time as the pulmonary function tests used in the analyses. Study participants were classified in two different ways: ever smokers vs never-smokers and current smokers vs never-smokers and past-smokers combined. Current smokers were defined as individuals who smoked at least one cigarette per day within the prior 12 months, past smokers were defined as smoking at least one cigarette per day but had stopped at least 12 months, and never smokers reported never having smoked. Ever smokers were a combination of current and past smokers.

### Genotyping and quality control

Genotyping of the discovery stage studies was performed mainly using the Illumina HumanExome BeadChip (Table S2). To improve accurate calling of rare variants, genotyped data from nine studies (ARIC, FHS, RS, CHS, AGES, LBC1936, and MESA) were called using GenCall in Illumina’s Genome Studio and the curated clustering files from the CHARGE joint calling effort^53^. The remaining studies (1958BC, KORA, and SHIP) called their genotypes in accordance to the UK exome chip consortium best practices^36^ using zCall^54^.

All studies performed comparable sample-level quality control steps and removed individuals according to the standard quality control metrics, i.e., sex mismatch, duplicate pairs, unexpected relatives, missing pulmonary function measurement, or missing covariate. Other than the standard variant-level quality control step^53^, we retained only nonsynonymous variants annotated by dbNSFP^26^ with MAF less than 5% among EAs and excluded monomorphic variants, variants with missing rates larger than 5%, and variants on the sex chromosomes. These low-frequency and rare variants were grouped by gene region in the aggregate variant tests^26^. Missing genotypes were imputed using a random draw from the binomial distribution with two trials and success probability equal to the estimated MAF. Genes with one or zero variants or overall cumulative minor allele count less than 20 were excluded.

The replication samples were genotyped using the Affymetrix UK BiLEVE or UK Biobank arrays, which both include substantial overlap with the Illumina Human Exome BeadChip^55^. Thorough sample and genotype quality control steps were undertaken before imputation to a combined 1000 Genomes^56^ and UK10K Project reference panel^57^. Following imputation, SNVs were excluded if they had imputation INFO score ≤ 0.5 or minor allele count < 3. Full details of the quality control and imputation procedure of the UK BiLEVE/UK Biobank genotype data were described elsewhere^52^.

### Statistical analysis

Since single-variant tests are usually underpowered for analyzing rare genetic variants, we used an aggregate variant test that analyzes multiple rare variants in a gene. For studies other than FHS, we utilized the R package rareGE^58^ to conduct gene-based interaction and joint tests that simultaneously test the genetic main effects as well as GxE interaction effects. As a set-based variance component test, rareGE has been shown to be powerful across different underlying GxE association patterns^19^. For FHS, we used a modified rareGE joint test which incorporates correlation among family members by including a random intercept with covariance structure proportional to the kinship matrix^19,59^. The rareGE interaction, joint and modified joint tests are briefly described in the Supplementary Materials. Both smoking variables were included in the linear regression model as covariates, while one of them was modeled as the primary exposure variable of interest and was tested for interactions. This resulted in a total of six pairs of pulmonary function traits (transformed FEV_1_, FVC, and ratio) and smoking status (ever vs never smoking or current vs past plus never smoking). Additional covariates included age, age-squared, sex, height, height-squared, recruitment sites, and the first 10 principal components accounting for any underlying population substructure. Principal components were constructed by each study using GWAS SNVs or exome-chip ancestry informative markers if GWAS not available^55^. Weight was only included as an adjustment variable for FVC as it is more strongly influenced by adiposity. Study-specific results were meta-analyzed by combining the *p*-values of the gene-based tests from each discovery study using Stouffer’s weighted Z-score method^60^: (1) transform *p*-values to z-values by the inverse CDF of a Gaussian distribution, i.e., $Z_{i} = {\Phi }^{ - 1} \left( {P_{i} } \right)$, (2) weight the z-values of each study by the sample size, i.e., $Z = \frac{{\mathop \sum \nolimits_{i = 1}^{k} w_{i} Z_{i} }}{{\sqrt {\mathop \sum \nolimits_{i = 1}^{k} w_{i}^{2} } }}$, where $w_{i} = \sqrt {N_{i} }$ and $N_{i}$ was the *i*th study’s sample size. The final meta-analysis *p*-value was calculated as $P = \phi \left( Z \right)$. Studies with genomic control inflation factor λ > 1.5 were excluded.

An a priori significance threshold was defined using the Bonferroni correction for the number of tests evaluated. Specifically, we used a significant threshold at 5.7E−07 based on 14,591 genes for 6 smoking status-lung outcome combinations. Significant genes were evaluated for replication in the UK BiLEVE and UK Biobank studies. To identify specific low-frequency or rare variants driving the signals from the aggregate variant tests, we conducted single variant analyses for the significant genes in both the discovery and replication datasets. Additionally, conditional analyses were performed for significant replicated genes (*GPR126* herein) with the previously reported leading common SNV, where the reported signal drivers were added in the gene-based test as covariates. For conditional analyses, *p*-values less than 0.05 were considered as nominally significant. We further investigated the tissue-specific gene expression of the significant genes on the GTEx Project portal (https://gtexportal.org/).

## Supplementary Information

Supplementary Information.
