# Supplementary material for: Rare and low-frequency exonic variants and gene-by-smoking interactions in pulmonary function
Source: Sci Rep. 2021 Sep 29;11:19365. doi: 10.1038/s41598-021-98120-7 (PMC8481467; doi:10.1038/s41598-021-98120-7)
Supplement: Supplementary file 1 — Supplementary Information. [file 41598_2021_98120_MOESM1_ESM.docx]

**Supplementary Materials for “Rare and Low-Frequency Exonic Variants and Gene-by-Smoking Interactions in Pulmonary Function”**

## Tianzhong Yang ^1,2^, Victoria E. Jackson ^3^,Albert V. Smith ^4^,Han Chen ^5,6^,Traci M. Bartz ^7,8^,Colleen M. Sitlani ^8^,Bruce M. Psaty ^9,10^,Sina A. Gharib ^11^,George T. O'Connor ^12,13^,Josée Dupuis ^14^,Jiayi Xu ^15,16^,Kurt Lohman ^17^,Yongmei Liu ^17^,Stephen B. Kritchevsky ^18^,Patricia A. Cassano ^16,19^,Claudia Flexeder ^20^,Christian Gieger ^21^,Stefan Karrasch ^20,22,23^,Annette Peters ^20,24^,Holger Schulz ^20,23^,Sarah E. Harris ^25,26^,John M. Starr ^26,27^,Ian J. Deary ^25,26^,Ani Manichaikul ^28,29^,Elizabeth C. Oelsner ^30,31^,R G. Barr ^31,32^,Kent D. Taylor ^33^,Stephen S. Rich ^34^,Tobias N. Bonten ^35^,Dennis O. Mook-Kanamori ^36,37^,Raymond Noordam ^38^,Ruifang Li-Gao ^36^,Marjo-Riitta Jarvelin ^39,40,41^,Matthias Wielscher ^39^,Natalie Terzikhan ^42,43^,Lies Lahousse ^42,43^,Guy Brusselle ^42,43^,Stefan Weiss ^44,45^,Ralf Ewert ^46^,Sven Gläser ^46,47^,Georg Homuth ^44^,Nick Shrine ^48^,Ian P. Hall ^49^,Martin Tobin ^48,50^,Stephanie J. London ^51*^,Peng Wei ^52*^,Alanna C. Morrison ^53*^

* Co-corresponding authors: Correspondence to Stephanie J. London ([london2@niehs.nih.gov](mailto:london2@niehs.nih.gov)), Peng Wei ([Pwei2@mdanderson.org](mailto:Pwei2@mdanderson.org)), and Alanna C. Morrison

([Alanna.C.Morrison@uth.tmc.edu](mailto:Alanna.C.Morrison@uth.tmc.edu)).

Affiliations

1.Department of Biostatistics and Data Science, School of Public Health, The University of Texas Health Science Center at Houston, Houston, TX, USA; 2.Division of Biostatistics, School of Public Health, University of Minnesota, Minneapolis, MN, USA; 3.Department of Health Sciences, University of Leicester, Leicester, UK; 4.Department of Biostatistics, University of Michigan, Ann Arbor, MI, USA; 5.Human Genetics Center, Department of Epidemiology, Human Genetics and Environmental Sciences, School of Public Health, The University of Texas Health Science Center at Houston, Houston, TX, USA; 6.Center for Precision Health, School of Public Health and School of Biomedical Informatics, The University of Texas Health Science Center at Houston, Houston, TX, USA; 7.Department of Biostatistics, University of Washington, Seattle, USA; 8.Cardiovascular Health Research Unit, Department of Medicine, University of Washington, Seattle, WA, USA; 9.Cardiovascular Health Research Unit, Departments of Medicine, Epidemiology and Health Services, University of Washington, Seattle, WA, USA; 10.Kaiser Permanente Washington Health Research Institute, Seattle, WA, USA; 11.Division of Pulmonary, Critical Care, and Sleep, Department of Medicine, University of Washington, Seattle, WA, USA; 12.Pulmonary Center, Department of Medicine, Boston University School of Medicine, Boston, MA, USA; 13.National Heart, Lung, and Blood Institute's Framingham Heart Study, Framingham, MA, USA; 14.Department of Biostatistics, Boston University School of Public Health, Boston, MA, USA; 15.Pamela Sklar Division of Psychiatric Genomics, Department of Genetics & Genomic Sciences, Icahn School of Medicine at Mount Sinai, New York, NY, USA; 16.Division of Nutritional Sciences, Cornell University, Ithaca, NY, USA; 17.Division of Cardiology, Department of Medicine, Duke Molecular Physiology Institute, Duke University School of Medicine, Durham, NC, USA; 18.Sticht Center for Healthy Aging and Alzheimer’s Prevention, Wake Forest School of Medicine, Winston-Salem, NC, USA; 19.Department of Healthcare Policy and Research, Weill Cornell Medical College, NY, USA; 20.Institute of Epidemiology, Helmholtz Zentrum München, German Research Center for Environmental Health, Neuherberg, Germany; 21.Research Unit of Molecular Epidemiology, Institute of Epidemiology, Helmholtz Zentrum München, German Research Center for Environmental Health, Neuherberg, Germany; 22.Institute and Outpatient Clinic for Occupational, Social and Environmental Medicine, Ludwig-Maximilians-Universität, Munich, Germany; 23.Comprehensive Pneumology Center Munich (CPC-M), Member of the German Center for Lung Research (DZL), Munich, Germany; 24.Institute for Medical Information Processing, Biometry and Epidemiology, Ludwig-Maximilians-Universität München, Munich, Germany; 25.Department of Psychology, The University of Edinburgh, Edinburgh, UK; 26.Centre for Cognitive Ageing and Cognitive Epidemiology, The University of Edinburgh, Edinburgh, UK; 27.Alzheimer Scotland Dementia Research Centre, The University of Edinburgh, Edinburgh, UK; 28.Center for Public Health Genomics, Department of Public Health Sciences, University of Virginia, Charlottesville, VA, USA; 29.Division of Biostatistics and Epidemiology, Department of Public Health Sciences, University of Virginia, Charlottesville,VA, USA; 30.Department of Medicine, College of Physicians and Surgeons, Columbia University, New York, NY, USA; 31.Department of Epidemiology, Mailman School of Public Health, Columbia University, New York, NY,USA; 32.Department of Medicine, Department of Epidemiology, Columbia University Medical Center, New York, NY,USA; 33.David Geffen School of Medicine, University of California, Los Angeles, Los Angeles, CA, USA; 34.Center for Public Health Genomics, Department of Public Health Sciences, University of Virginia, Charlottesville, VA, USA; 35.Department of Public Health & Primary Care, Leiden University Medical Center, Leiden, The Netherlands; 36.Department of Clinical Epidemiology, Leiden University Medical Center, Leiden, The Netherlands; 37.Department of Public Health & Primary Care, Leiden University Medical Center, The Netherlands; 38.Department of Internal Medicine, Division of Gerontology and Geriatrics, Leiden University Medical Center, Leiden, The Netherlands; 39.Dept of Epidemiology and Biostatistics, School of Public Health, Imperial College London, London, UK; 40.Center for Life Course Health Research, Faculty of Medicine, University of Oulu, Oulu, Finland; 41.Biocenter of Oulu, University of Oulu, Oulu, Finland.; 42.Department of Epidemiology, Erasmus University Medical Center, Rotterdam, The Netherlands; 43.Department of Respiratory Medicine, Ghent University Hospital, Ghent, Belgium; 44.Interfaculty Institute for Genetics and Functional Genomics, University Medicine Greifswald, Greifswald, Germany; 45.DZHK (German Centre for Cardiovascular Research), partner site Greifswald, Greifswald, Germany; 46.Department of Internal Medicine B, Division of Cardiology, Pneumology, Infectious Diseases, Intensive Care Medicine, University Medicine Greifswald, Greifswald, Germany; 47.Department of Internal Medicine, Vivantes Hospital Berlin Spandau, Berlin, Germany; 48.Department of Health Sciences, University of Leicester, Leicester, UK; 49.Division of Respiratory Medicine, University of Nottingham, Nottingham, UK; 50. National Institute for Health Research, Leicester Respiratory Biomedical Research Unit, Glenfield Hospital, Leicester, UK; 51.Epidemiology Branch, National Institute of Environmental Health Sciences, National Institutes of Health, Department of Health and Human Services, Research Triangle Park, NC, USA; 52.Department of Biostatistics, The University of Texas MD Anderson Cancer Center, Houston, TX, USA; 53.Human Genetics Center, Department of Epidemiology, Human Genetics and Environmental Sciences, School of Public Health, The University of Texas Health Science Center at Houston, Houston, TX, USA.

**Ethics statement**

The ARIC study has been approved by Institutional Review Boards (IRB) at all participating institutions: University of North Carolina at Chapel Hill IRB, Johns Hopkins University IRB, University of Minnesota IRB, and University of Mississippi Medical Center IRB. Study participants provided written informed consent at all study visits.

Written consent of the 1958BC was obtained from participants for the use of information in medical studies. The 45-year biomedical survey and genetic studies were approved by the South-East Multi-Centre Research Ethics Committee (ref: 01/1/44) and the joint UCL/UCLH Committees on the Ethics of Human Research. More information is available at <https://cls.ucl.ac.uk/wp-content/uploads/2017/07/NCDS-Ethical-review-and-Consent-2014.pdf>.

The FHS was reviewed and approved by the Boston University Medical Center IRB and informed consent was obtained from all participants.

Participants of HABC were recruited in the designated zip code areas surrounding Pittsburgh and Memphis. The institutional review boards at both sites approved the study.

The NFBC1966 have approved by the University of Oulu Ethnics Committee and the ethnical Committee of Northern Ostrobothnia Hospital District. Participants were provided written informed consent.

Written informed consent was obtained from all MESA study participants upon their arrival at the study clinic for each study examination. The institutional review boards of the six field centers and the National Heart, Lung and Blood Institute have approved the study protocol. In addition, the Institutional Review Board at the University of Virginia reviewed and approved our team’s access to MESA data for the analyses presented here.

The RS has been approved by the institutional review board (Medical Ethics Committee) of the Erasmus Medical Center (registration number MEC 02.1015) and by the Dutch Ministry of Health, Welfare and Sports (Population Screening Act WBO, license number 1071272-159521-PG). The approval has been renewed every 5 years, as well as with the introduction of major new elements in the study (e.g., MRI investigations). The Rotterdam Study Personal Registration Data collection is filed with the Erasmus MC Data Protection Officer under registration number EMC1712001. The Rotterdam Study has been entered into the Netherlands National Trial Register (NTR; [www.trialregister.nl](http://www.trialregister.nl)) and into the WHO International Clinical Trials Registry Platform (ICTRP; [www.who.int/ictrp/network/primary/en/](http://www.who.int/ictrp/network/primary/en/)) under shared catalogue number NTR6831. All participants provided written informed consent to participate in the study and to have their information obtained from treating physicians.

The CHS is a population-based cohort study of risk factors for cardiovascular disease in adults 65 years of age or older conducted across four field centers (NCT00005133 and NCT00149435). The CHS was approved by institutional review committees at each site and individuals in the present analysis gave informed consent including consent to use of genetic information for the study of cardiovascular disease.

The KORA-F4 study was approved by the ethics committee of the Bavarian Medical Association (EK Nr. 06068), and written informed consent has been given by all participants.

The AGES was approved by the National Bioethics Committee in Iceland that acts as the Institutional Review Board for the Icelandic Heart Association (approval number: VSN-00-063), and by the National Institute on Aging Intramural Institutional Review Board. A multistage consent is obtained in AGES to cover participation, use of specimens and DNA, and access to administrative records. All requests to merge AGES data with administrative, genealogic, hospital, or nationally maintained databases are reviewed by the Icelandic Data Protection Committee. Release of data for analysis is governed by rules created by these bodies to protect the privacy of Icelandic participants.

The NEO study was approved by the medical ethical committee of the Leiden University Medical Center (LUMC). Eligible participants were given detailed written information on the study, in addition to an oral explanation at the study site. Participants have given written informed consent for participation in the study, for storage of urine and blood samples, and for obtaining medical records and information on vital status during follow-up.

SHIP is a prospective longitudinal population-based cohort study in Mecklenburg-West Pomerania, Northern Germany assessing the prevalence and incidence of common diseases and their risk factors. All participants provided written informed consent. The study was approved by the ethics committee of the University Medicine Greifswald and carried out in accordance with its regulations.

Ethics permission for the Lothian Birth Cohort 1936 (LBC1936) was obtained from the Multi-Centre Research Ethics Committee for Scotland (MREC/01/0/56) and the Lothian Research Ethics Committee (LREC/2003/2/29). All participants gave informed consent.

The UK Biobank received ethics approval from the National Health Service

National Research Ethics Service (Ref 11/NW/0382, Ref (2016 renewal): 16/NW/0274). All participants provided written informed consent.

**Gene-based interaction only and joint test**

The gene-by-environment interaction model for p SNV is as follows:

$$E\left( Y_{i} | G_{i},E_{i}, X_{i} \right)=\sum_{j=1}^{p} {\alpha_{0j}G}_{ij}+\sum_{j=1}^{p} {\alpha_{1j}G}_{ij}E_{i}+\beta E_{i}+\theta X_{i},$$

where $Y_{i}$ is ranked-based inverse-normal transformed lung function trait for individual $i$, $G_{ij}$ the additive genotype for the j^th^ SNV in the gene, $E_{i}$and $X_{i}$ are the smoking variable and the covariate vector (including intercept) for individual *i*. $G_{ij}E_{i}$ is the interaction between the j^th^ SNV and smoking for individual i. $\alpha_{0j},\alpha_{1j}, \beta,$ and $\theta$ are the regression coefficients of the model. For the interaction only test, we test the null hypothesis that no SNV had interaction effects on $Y$, i.e.,$H_{0}: \alpha_{11}=\alpha_{12}=\ldots=\alpha_{1p}=0$ versus the alternative hypothesis ${H_{1}: \text{at least one} \alpha}_{1j}\neq0$. For joint test, we test whether at least one SNV had either main or interaction effects, i.e., $H_{0}: {\alpha_{01}=\ldots=\alpha_{0p}=\alpha}_{11}=\ldots=\alpha_{1p}$= 0 versus the alternative hypothesis $H_{1}: \text{at least one of}$ $\alpha_{0j}$ or $\alpha_{1j}\neq0.$ RareGE is a variance-component test, in which $\alpha_{11},\ldots,\alpha_{1p}$ are assumed to be random effects with mean 0 and covariance matrix $\tau_{1}I_{p\times p}$ and $\alpha_{01},\ldots,\alpha_{0p}$ are assumed to be random effects with mean 0 and covariance matrix $\tau_{0}I_{p\times p}$. It boils down to testing the variance parameters $\tau_{1}$ to be zero (interaction) and $\tau_{1}$ or $\tau_{0}$ to be zero (joint).

**Single SNP-based interaction and joint test**

When investigating a single SNP, we used the following model:

$$E\left( Y_{i} | G_{i},E_{i}, X_{i} \right)=\gamma_{0j}G_{ij}+\gamma_{1j}G_{ij}E_{i}+\beta E_{i}+\gamma X_{i},$$

For the interaction only test, we test whether the SNV had an interaction effect on $Y$ via Wald test, i.e.,$H_{0}:\gamma_{1j}$= 0. For joint test, we test whether the SNV had either a main or an interaction effect via likelihood ratio test, i.e., $H_{0}:\gamma_{1j}=\gamma_{0j}=0$.

**Revised rareGE for family-design studies**

The rareGE test was originally proposed for studies with independent samples (Chen, Meigs, & Dupuis, 2014a). To jointly test genetic main effect and gene-by-environment interaction for FHS with a family-based design, we modified the rareGE test with an additional random intercept. Specifically, we fitted a linear mixed model on quantitative outcomes under the null hypothesis, using a random intercept with covariance structure proportional to the kinship matrix to model correlation among family members (Chen, Meigs, & Dupuis, 2014b), then computed the variance component test statistic for the genetic main effects and gene-smoking interactions separately, and used a Monte Carlo method to compute the joint test p-value via numerical integration (Chen et al., 2014a)

| **** | **** |
| --- | --- |
| **** | **** |
| **** | **** |

Figure S1. Quantile-quantile plots for the six pairs of combinations of smoking variables and pulmonary function measures in the discovery stage.


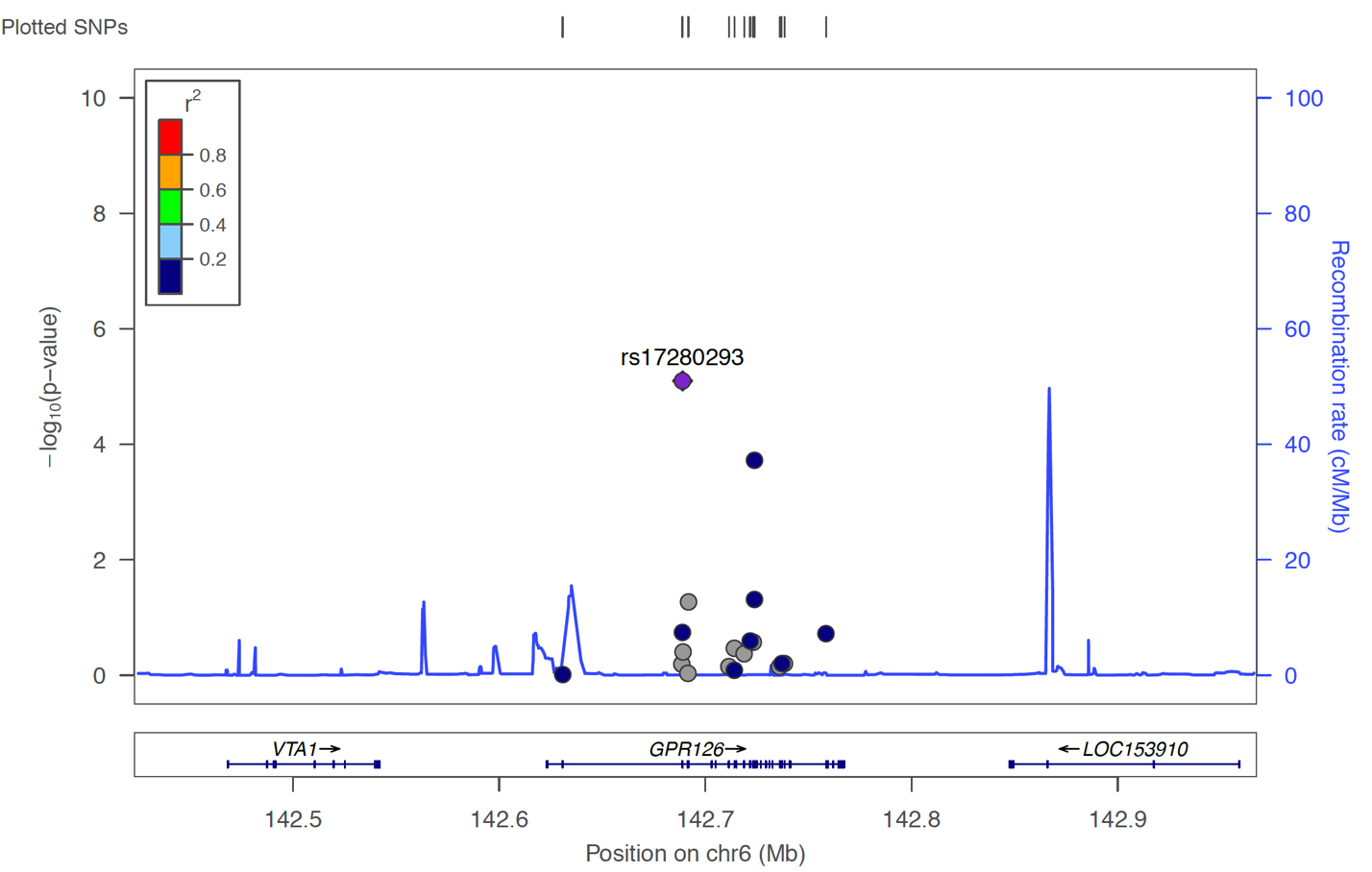


Figure S2. Single-variant based joint tests for *GPR126* (Current smoking vs FEV_1_/FVC ratio).


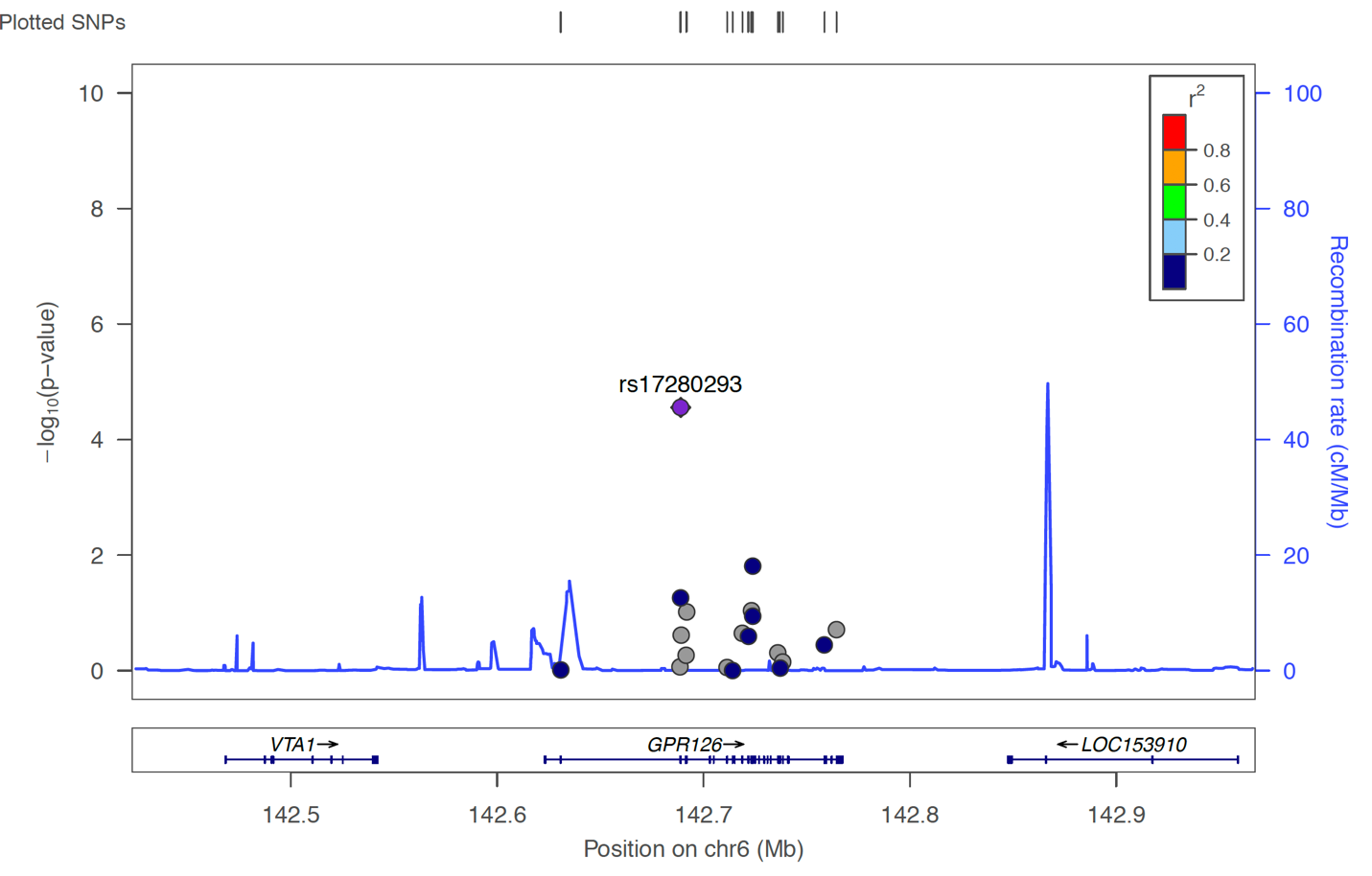


Figure S3. Single-variant based joint tests for *GPR126* (Ever smoking vs FEV_1_/FVC ratio).


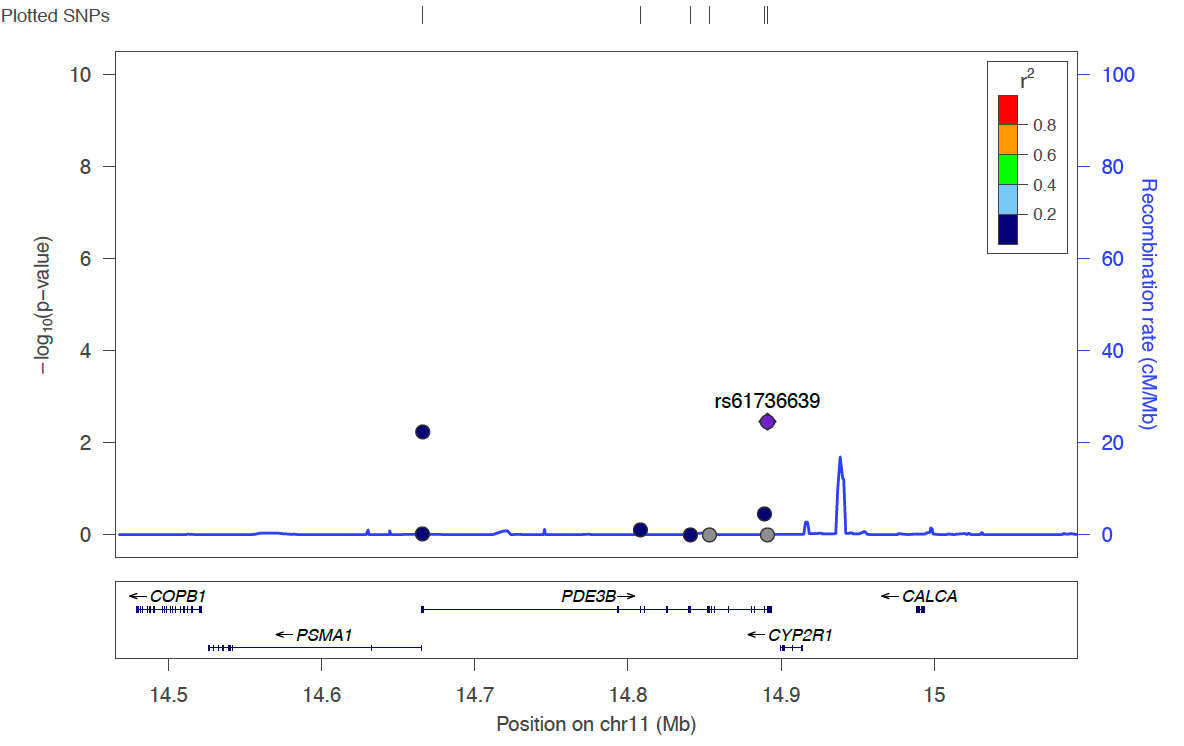


rs142935352

Figure S4. Single-variant based interaction tests for *PDE3B*. The leading rare variant rs61736639 is also within the range of *CYP2R1*.


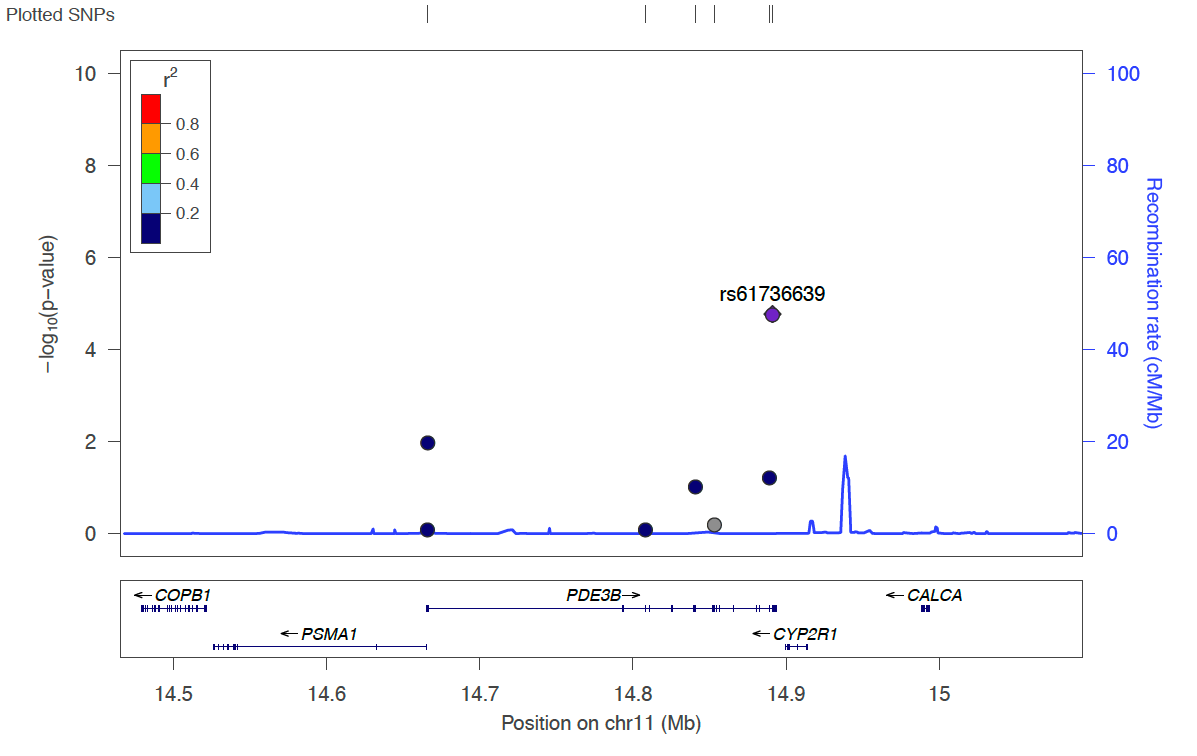
Figure S5. Single-variant based joint tests for *PDE3B*. The leading rare variant rs61736639 is also within the range of *CYP2R1*.

rs142935352

| Table S1. Study-specific Inflation factors for different combinations of the smoking status and spirometric measures of lung functions. Deleted studies are marked in boldface. | | | | |
| --- | --- | --- | --- | --- |
| **Smoking Status** | **Outcome** | **Study** | **Inflation factor (interaction)** | **Inflation factor (joint)** |
| Current | FVC | ARIC | 1.27 | 1.26 |
| Current | FVC | AGES | 1.27 | 1.18 |
| Current | FVC | 1958BC | 0.89 | 1.00 |
| Current | FVC | NFBC1966 | 1.12 | 1.10 |
| Current | FVC | RS | 0.94 | 1.08 |
| Current | FVC | SHIP | 0.85 | 1.03 |
| Current | FVC | FHS | NA | 1.15 |
| Current | FVC | CHS | 1.27 | 1.27 |
| Current | FVC | NEO | 1.12 | 1.16 |
| Current | FVC | HABC | 1.08 | 1.10 |
| Current | FVC | KORA | 1.34 | 1.19 |
| Current | FVC | LBC1936 | **1.59** | **1.55** |
| Current | FVC | MESA | 1.42 | 1.36 |
| Current | FEV1 | ARIC | 1.38 | 1.36 |
| Current | FEV1 | AGES | **1.53** | 1.32 |
| Current | FEV1 | 1958BC | 0.93 | 1.02 |
| Current | FEV1 | NFBC1966 | 1.18 | 1.11 |
| Current | FEV1 | RS | **1.51** | 1.45 |
| Current | FEV1 | SHIP | 0.96 | 1.07 |
| Current | FEV1 | FHS | NA | 1.21 |
| Current | FEV1 | CHS | 1.31 | 1.31 |
| Current | FEV1 | NEO | 1.15 | 1.10 |
| Current | FEV1 | HABC | 0.92 | 1.02 |
| Current | FEV1 | KORA | 1.35 | 1.19 |
| Current | FEV1 | LBC1936 | **1.82** | **1.69** |
| Current | FEV1 | MESA | **1.55** | 1.42 |
| Current | Ratio | ARIC | 1.26 | 1.24 |
| Current | Ratio | AGES | 0.93 | 0.98 |
| Current | Ratio | 1958BC | 1.13 | 1.16 |
| Current | Ratio | NFBC1966 | 0.91 | 0.96 |
| Current | Ratio | RS | 1.26 | 1.28 |
| Current | Ratio | SHIP | 1.03 | 1.12 |
| Current | Ratio | FHS | NA | 1.06 |
| Current | Ratio | CHS | 1.29 | 1.26 |
| **Smoking Status** | **Outcome** | **Study** | **Inflation factor (interaction)** | **Inflation factor (joint)** |
| Current | Ratio | NEO | 1.02 | 1.04 |
| Current | Ratio | HABC | 0.9 | 1.01 |
| Current | Ratio | KORA | 1.29 | 1.10 |
| Current | Ratio | LBC1936 | 1.16 | 1.23 |
| Current | Ratio | MESA | 1.46 | 1.42 |
| Ever | FVC | ARIC | 1.03 | 1.11 |
| Ever | FVC | AGES | 0.96 | 0.96 |
| Ever | FVC | 1958BC | 0.98 | 1.05 |
| Ever | FVC | NFBC1966 | 1.05 | 0.97 |
| Ever | FVC | RS | 0.84 | 1.05 |
| Ever | FVC | SHIP | 1 | 1.11 |
| Ever | FVC | FHS | NA | 1.02 |
| Ever | FVC | CHS | 0.95 | 1.06 |
| Ever | FVC | NEO | 0.93 | 1.01 |
| Ever | FVC | HABC | 0.94 | 1.03 |
| Ever | FVC | KORA | 0.99 | 0.98 |
| Ever | FVC | LBC1936 | 0.95 | 1.08 |
| Ever | FVC | MESA | 0.94 | 1.04 |
| Ever | FEV1 | ARIC | 1.08 | 1.16 |
| Ever | FEV1 | AGES | 0.96 | 0.99 |
| Ever | FEV1 | 1958BC | 0.96 | 1.01 |
| Ever | FEV1 | NFBC1966 | 1.08 | 1.02 |
| Ever | FEV1 | RS | 0.71 | 0.97 |
| Ever | FEV1 | SHIP | 0.97 | 1.03 |
| Ever | FEV1 | FHS | NA | 1.00 |
| Ever | FEV1 | CHS | 0.91 | 1.02 |
| Ever | FEV1 | NEO | 0.86 | 0.91 |
| Ever | FEV1 | HABC | 0.8 | 0.92 |
| Ever | FEV1 | KORA | 0.94 | 1.00 |
| Ever | FEV1 | LBC1936 | 0.94 | 1.07 |
| Ever | FEV1 | MESA | 0.87 | 0.98 |
| Ever | Ratio | ARIC | 0.98 | 1.06 |
| Ever | Ratio | AGES | 1.04 | 1.05 |
| Ever | Ratio | 1958BC | 1.04 | 1.10 |
| Ever | Ratio | NFBC1966 | 1.02 | 0.96 |
| Ever | Ratio | RS | 0.85 | 1.06 |
| **Smoking Status** | **Outcome** | **Study** | **Inflation factor (interaction)** | **Inflation factor (joint)** |
| Ever | Ratio | SHIP | 0.96 | 1.06 |
| Ever | Ratio | FHS | NA | 1.00 |
| Ever | Ratio | CHS | 0.96 | 1.05 |
| Ever | Ratio | NEO | 0.95 | 1.03 |
| Ever | Ratio | HABC | 0.87 | 0.97 |
| Ever | Ratio | KORA | 0.93 | 0.93 |
| Ever | Ratio | LBC1936 | 0.88 | 1.09 |
| Ever | Ratio | MESA | 0.96 | 1.17 |

Table S2. Genotyping array for the participating studies

| **Study** | **Genotyping platform** |
| --- | --- |
| ARIC | HumanExome BeadChip v1.0 |
| FHS | HumanExome BeadChip v1.0 |
| 1958BC | Illumina HiSeq 2500 |
| RS | HumanExome BeadChip v1.0 |
| SHIP | HumanExome BeadChip v1.0 |
| NFBC1966 | Illumina HiSeq 2000 |
| NEO | Illumina Infinium HumanCoreExome-12v1.0BeadChip |
| CHS | HumanExome BeadChip v1.0 |
| AGES | HumanExome BeadChip v1.0 |
| LBC1936 | HumanExome BeadChip v1.0 |
| KORA | HumanExome BeadChip v1.0 |
| MESA | HumanExome BeadChip v1.0 |
| HABC | HumanExome BeadChip v1.0 |
| UK BiLEVE | Affymetrix Axiom UK BiLEVE array |
| UK Biobank | Affymetrix Axiom UK Biobank array |

Table S3. Top 5 genes for gene-by-smoking interaction analysis. P-value is the meta-analyzed p-value from the interaction only test and cMAC is the mean of MAC across studies.

| **Smoking status** | **Outcome** | **Gene** | **P-value**  **(interaction)** | **cMAC** |
| --- | --- | --- | --- | --- |
| Current | FEV1 | *FATE1* | 1.09E-05 | 11.60 |
| Current | FEV1 | *C12orf66* | 3.16E-05 | 11.40 |
| Current | FEV1 | *PADI2* | 6.98E-05 | 70.59 |
| Current | FEV1 | *PDE3B* | 7.06E-06 | 132.44 |
| Current | FEV1 | *LCORL* | 7.26E-05 | 139.30 |
| Current | FVC | *GNA14* | 2.10E-05 | 55.81 |
| Current | FVC | *HCK* | 4.38E-05 | 175.81 |
| Current | FVC | *CCR2* | 6.66E-06 | 16.63 |
| Current | FVC | *ZDHHC12* | 8.93E-05 | 22.78 |
| Current | FVC | *ALDH3A1* | 9.38E-05 | 133.31 |
| Current | Ratio | *D4S234E* | 1.05E-05 | 51.43 |
| Current | Ratio | *GLB1L2* | 4.19E-05 | 389.62 |
| Current | Ratio | *MAN2B1* | 4.97E-05 | 204.34 |
| Current | Ratio | *SHROOM2* | 5.13E-05 | 167.90 |
| Current | Ratio | *SLCO5A1* | 6.81E-06 | 245.63 |
| Ever | FEV1 | *KIAA1147* | 1.45E-04 | 54.25 |
| Ever | FEV1 | *DCTD* | 1.66E-04 | 23.83 |
| Ever | FEV1 | *RASL12* | 1.85E-04 | 24.38 |
| Ever | FEV1 | *MYO18A* | 4.48E-05 | 120.84 |
| Ever | FEV1 | *NOM1* | 5.21E-05 | 115.08 |
| Ever | FVC | *NPAS4* | 1.03E-04 | 90.85 |
| Ever | FVC | *CXorf30* | 1.05E-04 | 5.67 |
| Ever | FVC | *TTC3* | 1.84E-04 | 772.25 |
| Ever | FVC | *ANO2* | 2.86E-04 | 148.24 |
| Ever | FVC | *WDR54* | 2.98E-04 | 74.91 |
| Ever | Ratio | *ASXL2* | 1.44E-04 | 209.54 |
| Ever | Ratio | *KARS* | 1.53E-04 | 64.08 |
| Ever | Ratio | *FAM98A* | 1.78E-04 | 145.40 |
| Ever | Ratio | *ZNF527* | 1.97E-04 | 5.33 |
| Ever | Ratio | *ARHGEF33* | 2.30E-04 | 173.11 |

Table S4. Top 5 genes for gene-by-smoking interaction analysis. P-value is the meta-analyzed p-value from the joint test and cMAC is the mean of MAC across studies.

| **Smoking status** | **Outcome** | **Gene** | **P-value**  **(joint)** | **cMAC** |
| --- | --- | --- | --- | --- |
| Current | FEV1 | *FATE1* | 2.36E-06 | 11.60 |
| Current | FEV1 | *PDE3B* | 2.93E-07 | 132.44 |
| Current | FEV1 | *ASNSD1* | 4.89E-05 | 99.25 |
| Current | FEV1 | *CCDC18* | 4.99E-05 | 204.71 |
| Current | FEV1 | *RBM41* | 6.23E-06 | 12.29 |
| Current | FVC | *RPAP2* | 1.37E-05 | 53.00 |
| Current | FVC | *PDP2* | 1.91E-05 | 36.00 |
| Current | FVC | *ZNF180* | 2.75E-05 | 209.70 |
| Current | FVC | *C8orf85* | 6.30E-05 | 18.00 |
| Current | FVC | *GABRG3* | 7.67E-05 | 164.25 |
| Current | Ratio | *GPR126* | 1.89E-09 | 263.84 |
| Current | Ratio | *GRIK4* | 3.45E-05 | 91.09 |
| Current | Ratio | *ATP13A2* | 4.31E-05 | 176.46 |
| Current | Ratio | *PDE9A* | 5.55E-05 | 113.15 |
| Current | Ratio | *D4S234E* | 7.63E-05 | 51.43 |
| Ever | FEV1 | *MYO18A* | 2.44E-05 | 120.84 |
| Ever | FEV1 | *ZSCAN10* | 2.86E-05 | 80.61 |
| Ever | FEV1 | *ZNF736* | 4.38E-05 | 64.60 |
| Ever | FEV1 | *GPR156* | 4.61E-05 | 75.42 |
| Ever | FEV1 | *HLA-DQA2* | 8.81E-05 | 191.63 |
| Ever | FVC | *ACTL7B* | 1.15E-04 | 57.67 |
| Ever | FVC | *NFATC1* | 1.29E-04 | 54.11 |
| Ever | FVC | *GPR18* | 1.29E-04 | 60.11 |
| Ever | FVC | *RPAP2* | 1.79E-05 | 53.00 |
| Ever | FVC | *PDP2* | 9.30E-05 | 36.00 |
| Ever | Ratio | *ARHGEF33* | 1.28E-04 | 173.11 |
| Ever | Ratio | *PCDHA10* | 1.92E-04 | 16.50 |
| Ever | Ratio | *CALML5* | 1.81E-05 | 55.80 |
| Ever | Ratio | *GPR126* | 2.76E-08 | 263.84 |
| Ever | Ratio | *ITGA7* | 5.59E-05 | 196.77 |

**Reference**

Chen, H., Meigs, J. B., & Dupuis, J. (2014a). Incorporating gene-environment interaction in testing for association with rare genetic variants. *Human Heredity*, *78*(2), 81–90. https://doi.org/10.1159/000363347

Chen, H., Meigs, J. B., & Dupuis, J. (2013). Sequence Kernel Association Test for Quantitative Traits in Family Samples. *Genetic Epidemiology*, *37*(2), 196–204.
